# The complete mitochondrial genome of *Nycteribia formosana* (Diptera, Nycteribiidae)

**DOI:** 10.1080/23802359.2023.2290127

**Published:** 2023-12-18

**Authors:** Xiaoyan Zheng, Xiaobin Huang, Jinting Yang, Huijuan Yang, Xianzheng Zhang

**Affiliations:** Institute of Pathogens and Vectors, Yunnan Provincial Key Laboratory for Zoonosis Control and Prevention, Dali University, Dali, China

**Keywords:** Phylogeny, mitochondrial genome, *Nycteribia formosana*

## Abstract

The family Hippoboscidae is an ectoparasite that primarily inhabits bats and relies on the host’s blood for sustenance. This research provides the first complete mitochondrial genome of *Nycteribia formosana*, which shares similar characteristics with other dipteran insects. The circularized mitochondrial genome, spanning 15,107 bp, encompasses 13 protein-coding genes (PCGs), 22 transfer RNA genes (tRNA), two ribosomal RNA genes, and a control region. The nucleotide composition of A, C, G, and T is 40.4%, 10.9%, 6.7%, and 42.0%, respectively. The findings from the phylogenetic analysis suggest that the species under investigation forms a cluster with other species belonging to the family Nycteribiidae. Consequently, this study provides valuable insights for the identification of *N. formosana*.

## Introduction

The family Nycteribiidae, which falls under the order Diptera and the superfamily Hippoboscoidea, consists of highly specialized blood-feeding flies that exclusively associate with bats (Mammalia: Chiroptera). These flies, known as bat flies along with the family Streblidae, are obligate body surface parasites of bats. They parasitize the host’s fur and wing membranes, primarily feeding on the host’s blood (Dick and Dittmar 2013). Nycteribiids exhibit distinct morphological features: this group is characterized by their complete lack of wing and spider-like appearance, the thorax is dorsoventrally flattened, head size is reduced, and eyes are either reduced or completely absent (Dick and Patterson 2006).

Bats are widely believed to serve as the primary source of transmission for numerous zoonotic pathogens. Among these pathogens, bat flies have been identified as potential carriers and significant contributors to the transmission and persistence of bat borne diseases. In recent decades, a substantial number of pathogens have been detected in bats and their ectoparasites on a global scale (Han et al. 2021), including *Bartonella* (McKee et al. 2021), *haemosporidian* (Sándor et al. 2021), *Rickettsia*, Mahlapitsi virus (Szentiványi et al. 2019), and others. The presence of these pathogens poses a serious threat to human safety.

Based on the available data, *Nycteribia formosana* (Karaman, 1939) is predominantly a parasite of the genus *Myotis* and was initially detected in Taiwan, China (Orlova et al. 2021). Other documented occurrences of this species have been reported in Korea and Russia (Mogi et al. 2023). Notably, *N. formosana* shares striking morphological similarities with *Nycteribia uenoi*, thereby posing difficulties in their taxonomic differentiation (Kim et al. 2012). Consequently, the accurate identification of *N. formosana* presents a considerable challenge. Thus, in light of these findings, our study has contributed to the comprehensive characterization of the mitochondrial genome of *N. formosana*, enhanced the knowledge regarding species within the Nycteribiidae family, and established a solid genomic resource base for future investigations.

## Materials

### Sample collection

*N. formosana* samples were obtained from *Myotis fimbriatus* individuals captured in Shibao Mountain (26°24′13″N, 99°50′31″E) in Jianchuan County, Dali City, Yunnan Province, China. These samples were subsequently identified by Xiaoyan Zheng through microscopic examination of their morphological characteristics. Following identification, individual *N. formosana* specimens were carefully prepared and preserved at the Pathogen and Vector Biology Research Institute of Dali University (website: https://www.dali.edu.cn/kxyj/yjs/1611.htm; contact person: Xiaobin Huang, huangxb633@nenu.edu.cn) under the voucher number DLBC2022001. The samples were stored at 95% ethanol and −20 °C.

### Statement

The capture, procedures, and handling of *Myotis fimbriatus* were conducted in accordance with the regulations and guidelines established by the Ethics Committee of Dali University, under the approval number MECDU-202104-27. This study adhered rigorously to ethical standards and guidelines pertaining to animal handling.

## Methods

### DNA extraction, mitogenome sequencing, and annotation

First, the genomic DNA of *N. formosana* was extracted using the Tissue DNA Kit (Omega, Norcross, GA). The experiment utilized a single individual to complete the genome assembly. The complete mitochondrial genome of *N. formosana* was sequenced using the Illumina Novoseq 6000 sequencing platform. The type of flowcell used for Illumina is MiSeq flowcell, which has a read length of 150,150 and is a paired read. In this particular study, the MitoZ 2.3 software (https://doi.org/10.1093/nar/gkz173) was employed for the assembly of the mitochondrial genome (Meng et al. 2019), while MITOS (Bernt et al. 2013) was utilized for annotation purposes. Lastly, a circular mitochondrial genome map was generated using the online platform GenomeVx (Conant and Wolfe 2008). The nucleotide sequence of *N. formosana* has been deposited in the GenBank database under the accession number OQ675011.

### Sequence annalysis

The nucleotide composition of *N. formosana* was analyzed using Geneious Prime software (Kearse et al. 2012). *Chironomus tepperi* (Beckenbach 2012) and *Dixella aestivalis* (Briscoe et al. 2015) were utilized as outgroups in the construction of the phylogenetic tree. The phylogenetic tree, emcompassing *N. formosana* and 14 other species previously published on NCBI, was constructed using the IQ-TREE (Trifinopoulos et al. 2016) network server, based on the nucleotide sequences of 13 protein-coding genes (PCGs). The best-fit models for maximum-likelihood (ML) were determined through the utilization of Modelfinder (Kalyaanamoorthy et al. 2017). The ML phylogenetic tree was constructed employing 1000 ultrafast bootstraps under the GTR + F + I + G4 model, while support for evolutionary branches was assessed by means of a non-parametric bootstrap method with 1000 replicates. The GenBank accession numbers corresponding to the 14 species in question are provided: *Basilia ansifera* MZ826150 (Porter et al. 2022), *Dipseliopoda setosa* MZ826151 (Porter et al. 2022), *Glossina austeni* MZ826152 (Porter et al. 2022), *Glossina brevipalpis* MZ826153 (Porter et al. 2022), *Lipoptena* sp. MT679542 (Wang et al. 2021), *Melophagus ovinus* KX870852 (Liu et al. 2017), *Ornithomya biloba* MZ379837 (Li et al. 2022), *Paradyschiria parvula* MK896865 (Trevisan et al. 2019), *Paratrichobius longicrus* MK896866 (Trevisan et al. 2019), *Brachytarsina amboinensis* OQ301749 (Poon et al. 2023), *Raymondia* sp. OQ301747 (Poon et al. 2023), *Phthiridium* sp. OQ301748 (Poon et al. 2023), *Phthiridium szechuanum* OP459298 (Zhang et al. 2023), and *Nycteribia parvula* OP442519 (Yang et al. 2023).

## Results and discussion

### General characteristics of the mitochondrial genome

**Figure 1.**
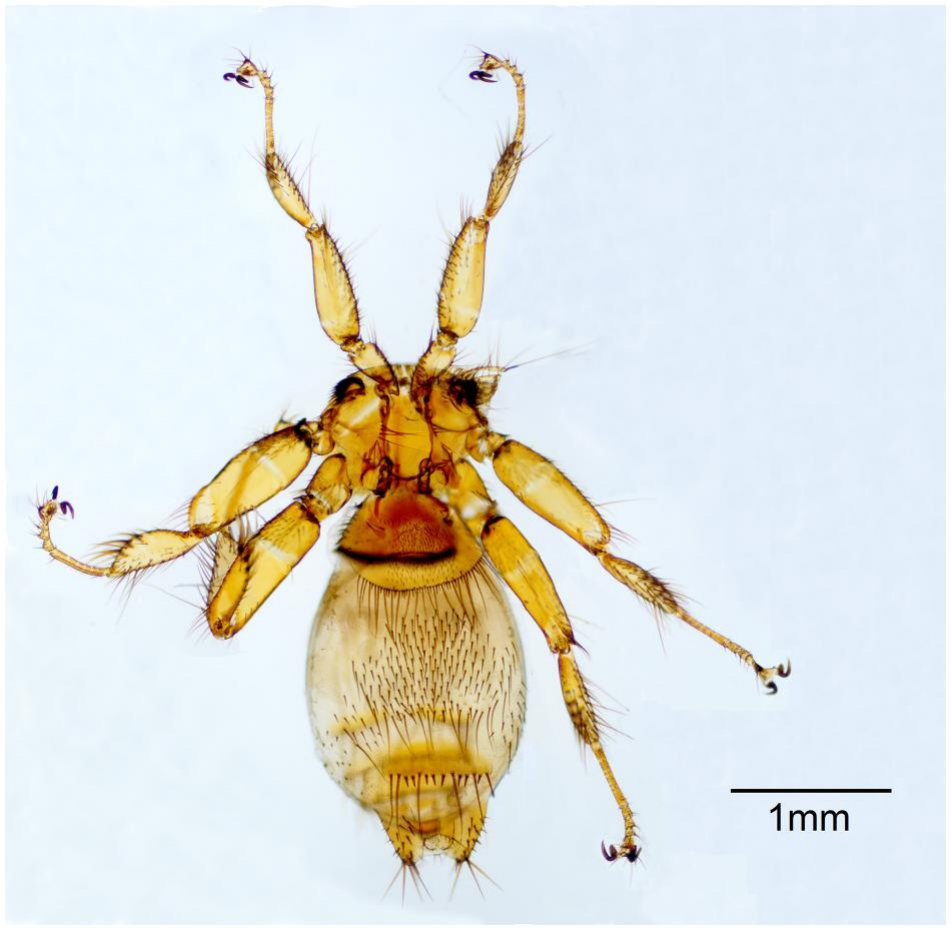
Representative of *N. formosana* collected in this study (the photo was taken by Xiaoyan Zheng).

The circular mitochondrial genome of *N. formosana* was found to have a total length of 15,107 bp (GenBank accession number: OQ675011). This genome comprises 37 mitochondrial genes, including the standard 13 PCGs, 22 tRNA genes, two rRNA genes, and a control region. The sequence data obtained from this genome aligns with earlier investigations conducted on Diptera genomes (Porter et al. 2022). In the 15,107 bp nucleotide sequence of *N. formosana*, the distribution of nucleotides is as follows: A = 6097 (40.4%), C = 1649 (10.9%), G = 1012 (6.7%), and T = 6349 (42.0%). The combined content of A + T is notably high at 82.4%, significantly surpassing the content of G + C (17.6%). Among the 13 PCGs analyzed, it was observed that five PCGs (*nad2*, *atp8*, *nad3*, *nad4L*, *nad6*) commence with the initiation codon ATT, while another set of five PCGs (*cox2*, *atp6*, *cox3*, *nad4*, *cytb*) initiate with the codon ATG. On the other hand, *cox1*, *nad5*, and *nad1* initiate with the codons TCG, TTG, and TTG, respectively. Furthermore, seven PCGs (*nad2*, *cox2*, *atp8*, *atp6*, *cox3*, *nad4L*, *nad6*) terminate with the codon TAA, while 3 PCGs (*nad1*, *cytb*, *nad3*) terminate with the codon TAG. Interestingly, *cox1*, *nad5*, and *nad4* exhibit an incomplete stop codon, terminating with a single T residue, the remaining 22 transfer RNA (tRNA) sequences and two ribosomal RNA (rRNA). Sequences exhibit varying lengths, ranging from 62 bp to 1365 bp (Figure 1).

### Phylogenetic analysis

**Figure 2.**
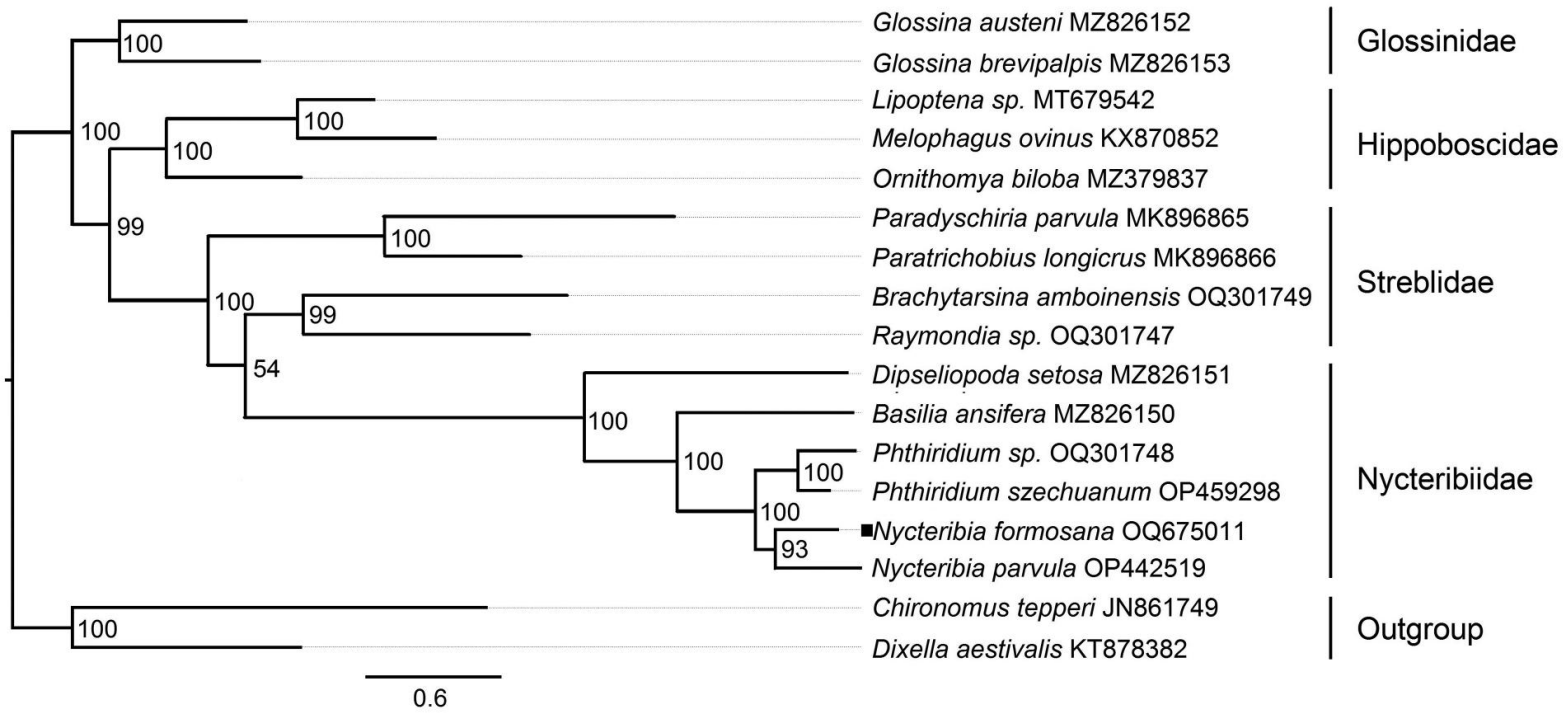
The maximum-likelihood phylogenetic tree of 15 species was inferred by IQ-TREE based on nucleotide sequence of 13 protein-coding genes. ‘Square’ indicates newly sequenced data in this study. The vertical row indicates species of the same family.

**Figure 3.**
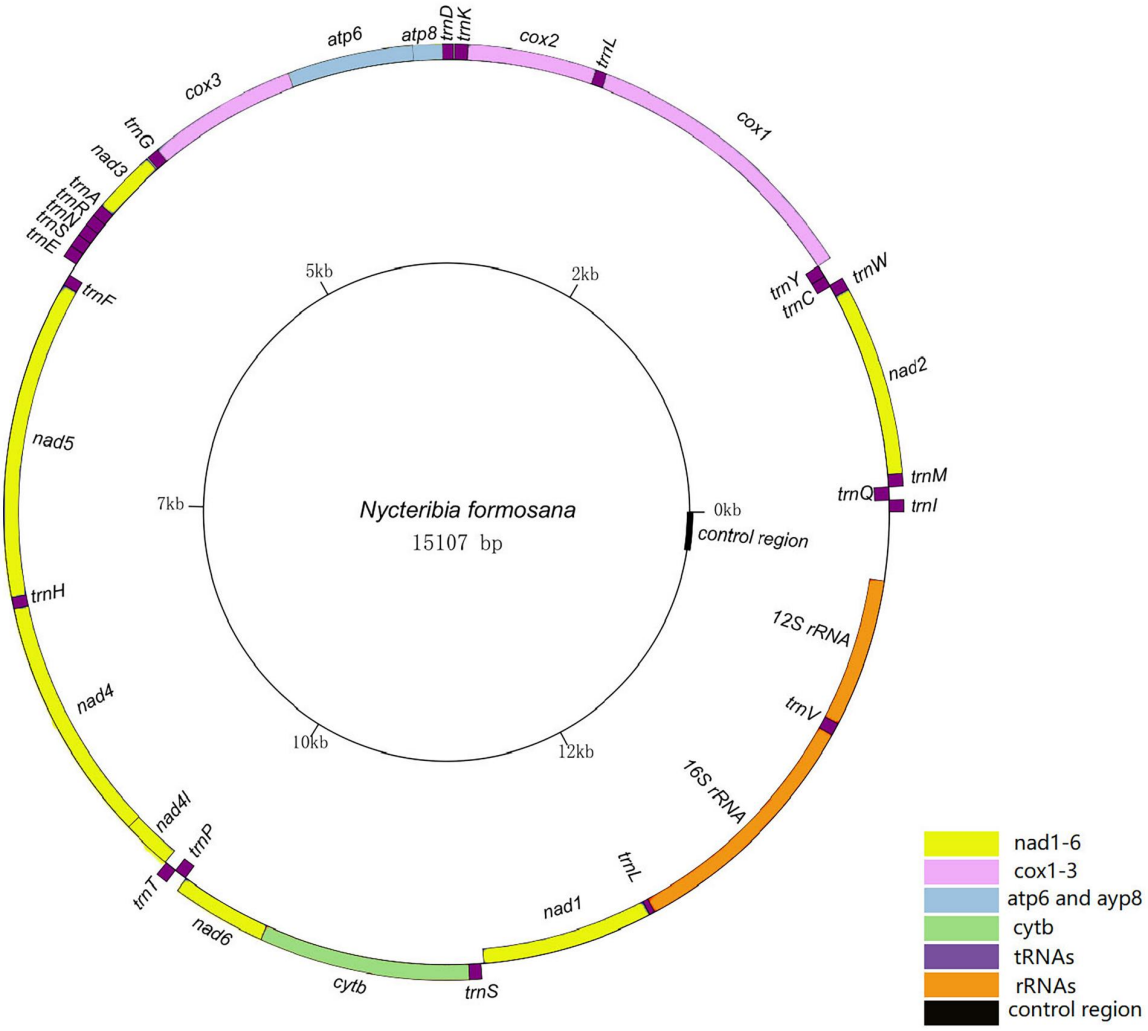
The circular mitochondrial genome map of *N. formosana.*

According to the phylogenetic tree depicted in Figure 2, *N**. formosana*, *Dipseliopoda setosa*, *Basilia ansifera*, *Phthiridium szechuanum*, and *Nycteribia parvula* form a single clade for the family Nycteribiidae. The molecular phylogeny supports the placement of *N. formosana* in the family Nycteribiidae, and the close proximity of *Nycteribia parvula* provides evidence for the genus identification (Figure 3).

## Discussion and conclusions

This study presents a comprehensive analysis of the gene sequence of *N. formosana*, marking the first investigation for this species. The complete mitochondrial genome information of this species was successfully obtained. Furthermore, the phylogenetic analysis provided additional evidence supporting the homology between *N. formosana* and other related species. Consequently, this study effectively addresses the present limited amount of molecular data on *N. formosana*, thereby furnishing crucial DNA molecular information for prospective research endeavors encompassing phylogenetic studies, evolutionary analysis, and species identification within the bat flies.

## Supplementary Material

Supplemental Material

Supplemental Material

## Data Availability

The genomic sequence data supporting this study can be accessed from GenBank on the NCBI website (https://www.ncbi.nlm.nih.gov/) with reference number OQ675011. The associated SRA, BioSample, and BioProject numbers are SRR23951134, SAMN33858843, and PRJNA947516, respectively.
