# Supplementary figures and images for: The complete mitochondrial genome of Nycteribia formosana (Diptera, Nycteribiidae)
Source: Mitochondrial DNA B Resour. 2023 Dec 18;8(12):1406–10. doi: 10.1080/23802359.2023.2290127 (PMC10732201; doi:10.1080/23802359.2023.2290127)

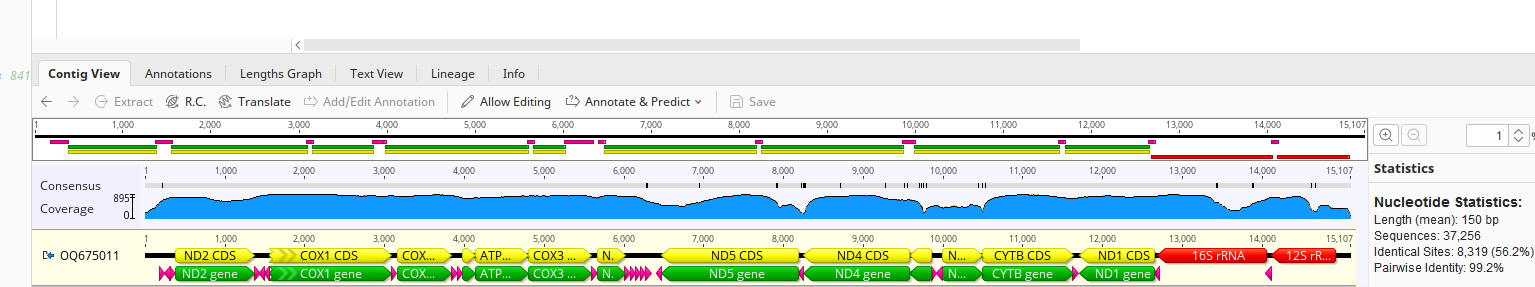

Supplement: Supplemental Material [file TMDN_A_2290127_SM6615.jpg]
