# Supplementary material for: The complete mitochondrial genome of Nycteribia formosana (Diptera, Nycteribiidae)
Source: Mitochondrial DNA B Resour. 2023 Dec 18;8(12):1406–10. doi: 10.1080/23802359.2023.2290127 (PMC10732201; doi:10.1080/23802359.2023.2290127)
Supplement: Supplemental Material [file TMDN_A_2290127_SM6614.pdf]

大理大学
DALI UNIVERSITY

大理大学医学伦理委员会
Medical Ethics Committee of Dali University(MECDU)

# 医学伦理审查批准书

## Affidavit of Approval of Medical Ethics and Welfare

| 批准编号 Approval No. | MECDU-202104-27 |
|---|---|

本《实验方案》经过大理大学医学伦理委员会审核，符合医学实验伦理原则，符合国家医学实验伦理福利的相关规定。实验方案的相关信息如下：

This Experimental Protocol has been reviewed by the Medical Ethics Committee of Dali University and conforms to the principles of medical experiment ethics and the relevant provisions of the national medical experiment ethics and welfare. The relevant information of the experimental program is as follow:

| | | | | | |
|---|---|---|---|---|---|
| 课题名称 Protocol Title | 云南省中华菊头蝠体表寄生虫多样性及影响因素研究<br>Research on The Diversity of Ectoparasites and Related Factors on *Rhinolophus sinicus* in Yunnan Province | | | | |
| 申请人 Applicant | 黄晓宾<br>Xiaobin Huang | 职称/学位 Title/Degree | 助理研究员/博士<br>Assistant Researcher/doctor | 邮箱 Email | huangxb633@nenu.edu.cn |
| 课题负责人 Principle Investigator (PI) | 黄晓宾<br>Xiaobin Huang | 职称/学位 Title/Degree | 助理研究员/博士<br>Assistant Researcher/doctor | 邮箱 Email | huangxb633@nenu.edu.cn |
| 院系(部门) Institution | 大理大学/病原与媒介生物研究所<br>Dali university/Institute of pathogens and vectors | | | 申请日期 Application date | 2021年04月18日 |
| 实验主要内容 experimental design | 探究云南省中华菊头蝠体表寄生虫多样性以及影响多样性的主要因素。<br>The present study was designed to investigate the species diversity of ectoparasites and related main factors on *R. sinicus* in Yunnan Province | | | | |
| 计划执行时间 Period of Protocol | 2021年9月——2023年8月 | | | | |
| 审查意见 Results of inspection | ☑符合医学伦理福利要求，可以进行实验 Approval<br>☐调整方案后，可以进行实验 Resubmit after modification<br>☐不符合医学伦理福利要求，不可以进行实验 Rejection | | | | |

大理大学医学伦理委员会

Medical Ethics Committee of Dali University(Seal)

日期 Date　2021.04.18

地址：中国，云南省大理市，古城弘圣路 2 号，大理大学，邮编：671003，电话：0872-2219936
Add: NO.2 Hongsheng Road, Dali, Yunan 671003 P.R. CHINA, 671003，Tel: 0872-2219936
